# Recruitment and Baseline Characteristics of Participants in the Social, Emotional, and Economic Empowerment Through Knowledge of Group Support Psychotherapy Study (SEEK-GSP): Cluster Randomized Controlled Trial

**DOI:** 10.2196/11560

**Published:** 2019-01-03

**Authors:** Etheldreda Nakimuli-Mpungu, Seggane Musisi, Kizito Wamala, James Okello, Sheila Ndyanabangi, Josephine Birungi, Mastula Nanfuka, Michael Etukoit, Ramin Mojtabai, Jean Nachega, Ofir Harari, Edward Mills

**Affiliations:** 1 Department of Psychiatry College of Health Sciences Makerere University Kampala Uganda; 2 Center for Victims of Torture Department of Psychology Gulu Uganda; 3 Department of Mental Health Faculty of Medicine Gulu University Gulu Uganda; 4 Mental Health Program Ministry of Health of Uganda Kampala Uganda; 5 The AIDS Support Organization (TASO) Kampala Uganda; 6 Department of Mental Health Bloomberg's School of Public Health Johns Hopkins University Baltimore, MD United States; 7 Department of Epidemiology Pittsburg Graduate School of Public Health University of Pittsburg Pittsburgh, PA United States; 8 Stellenbosch Center for Infectious Disease Department of Medicine Stellenbosch University Cape Town South Africa; 9 Department of Epidemiology Bloomberg's School of Public Health Johns Hopkins University Baltimore, MD United States; 10 Department of International Health Bloomberg's School of Public Health Johns Hopkins University Baltimore, MD United States; 11 MTEK Sciences Inc Vancouver, BC Canada; 12 Department of Clinical Epidemiology & Biostatistics McMaster University Hamilton, ON Canada

**Keywords:** cluster randomized trial, group support psychotherapy, lay health workers, depression, recruitment, psychosocial stressors, persons living with HIV/AIDS, Uganda

## Abstract

**Background:**

Psychosocial characteristics, including self-esteem, perceived social support, coping skills, stigma, discrimination, and poverty, are strongly correlated with depression symptoms. However, data on the extent of these correlations among persons living with HIV and the associations between psychosocial characteristics and HIV treatment outcomes are limited in sub-Saharan Africa.

**Objective:**

This paper aims to describe the recruitment process and baseline characteristics associated with depression in a sample of HIV-positive people in a cluster randomized trial of group support psychotherapy (GSP) for depression delivered by trained lay health workers (LHWs).

**Methods:**

Thirty eligible primary care health centers across three districts in Uganda were randomly allocated to have their LHWs trained to deliver GSP (intervention arm) or group HIV education and treatment as usual (control arm) to persons living with HIV comorbid with depression. Baseline demographic, socioeconomic, and psychosocial characteristics were collected via interviewer-administered questionnaires. Among eligible participants, differences between those enrolled versus those who refused enrollment were assessed using chi square for categorical variables and t tests for continuous variables. Spearman rank order correlation analyses were conducted to determine associations between baseline depression symptoms and adherence to antiretroviral therapy (ART), viral load suppression, and other psychosocial variables.

**Results:**

The study screened 1473 people and 1140 were found to be eligible and enrolled over 14 weeks. Participants recruited comprised 95% of the target sample size of 1200. The sample’s mean age was 38.5 (SD 10.9) years and both genders were well represented (males: 46.32%, 528/1140). Most participants met the diagnostic criteria for major depressive disorder (96.92%, 1105/1140), had significant posttraumatic stress symptoms (72.46%, 826/1140), reported moderate suicide risk (52.54%, 599/1140), had primary or no formal education (86.22%, 983/1140), and reported no income-generating activity (72.63%, 828/1140) and no food insecurity (81.67%, 931/1140). Among eligible participants, 48 of 1140 (4.21%) refused to participate in the interventions; these participants were more likely to be males (χ^2^_1_=4.0, *P*=.045) and have significantly lower depression symptoms scores (t_2_=2.36, *P*=.01) than those who participated in the interventions. There was a significant positive correlation between viral load and number of traumatic experiences (ρ=.12, *P*=.05). Adherence to ART was positively correlated with perceived social support (ρ=.15, *P*<.001), but negatively correlated with depression symptoms (ρ=–.11, *P*=.05) and stigma (ρ=–.14, *P*<.001).

**Conclusions:**

Men and women with HIV and depression experience multiple social and economic vulnerabilities and disadvantages. Culturally tailored psychological interventions aimed at these individuals should address these socioeconomic disadvantages in addition to addressing their mental health care needs.

**Trial Registration:**

Pan African Clinical Trials Registry PACTR201608001738234; https://pactr.samrc.ac.za/TrialDisplay.aspx?TrialID=1738 (Archived by WebCite at http://www.webcitation.org/74NtMphom)

## Introduction

Individuals with major depression account for a substantial proportion of antiretroviral therapy (ART) users attending HIV treatment centers in sub-Saharan Africa. Yet they hardly receive any mental health services [1,2]. Several studies in HIV-positive populations both in developed and developing countries have shown that psychosocial stressors, such as a lack of social support and poor coping skills [3,4], low self-esteem [5], lack of financial resources [6], and internalized stigma and discrimination [7,8] are associated with depression. Furthermore, several studies conducted in HIV-positive populations have shown that alcohol use problems and posttraumatic stress are frequent and often complicate depression [9,10]. However, data on the extent of these correlations among persons living with HIV in sub-Saharan Africa are limited.

Past research studies in developed countries have shown that depression and related psychosocial stressors influence progression of HIV disease [11]. For example, there is some evidence that persons living with HIV (PLWH) who report experiencing stigma and discrimination have worse health outcomes [12]. Past research has also shown that lack of social support and poor coping skills [13], poor financial resources [14], and food insecurity [15] among ART users have been associated with poor HIV treatment outcomes. However, most past research on these associations come from high-income countries and from studies with small sample sizes. The correlations between psychosocial characteristics and HIV treatment outcomes in other parts of the world, and especially in sub-Saharan Africa where HIV is highly prevalent, are unknown.

Further, although studies of psychological interventions with depressed PLWH in high-income countries have endeavored to document related psychosocial stressors or HIV treatment outcomes [16], those conducted in low- and middle-income countries have rarely done so [17]. As a result, there is little information on the psychosocial stressors associated with HIV treatment from low- and middle-income countries. This makes it difficult to understand and appreciate the social context of PLWH in these settings. Past research from the United States has highlighted the role of various environmental stressors related to poverty, persistent residential mobility, racial discrimination, and inadequate access to resources on HIV care and outcomes. These findings indicate the importance of attending to social context in addition to clinical factors in planning interventions for PLWH [18].

To attend to the contextual realities of PLWH in sub-Saharan Africa, we developed group support psychotherapy (GSP)—a culturally sensitive cognitive behavioral-based intervention that treats depression by enhancing emotional and social support, positive coping, and livelihood skills [19]. The Social, Emotional, and Economic empowerment through Knowledge of GSP (SEEK-GSP) trial follows a series of pilot studies [20,21] that demonstrated the effectiveness of GSP in treating mild to moderate depression. This trial will provide robust evidence for the change processes and outcomes we observed in these pilot studies.

The PLWH participating in the SEEK-GSP trial were recruited from a postconflict region where there is a heavy burden of depression, posttraumatic stress symptoms, and psychosocial stressors [22]. This sample provides an opportunity to understand the processes and challenges in recruiting this highly vulnerable population, who are rarely recruited into clinical trials. The purpose of this paper is to summarize the recruitment procedures and baseline results of this trial, and explore associations among depression symptoms, related psychosocial stressors, adherence to ART, and viral suppression.

## Methods

### Overview of Study Design and Interventions

A detailed description of the study protocol is published elsewhere [23] and is registered in the Pan African Clinical Trials Registry (PACTR201608001738234). Briefly, this is a pragmatic two-arm cluster randomized trial evaluating the effectiveness GSP delivered by trained lay health workers (LHWs) to PLWH presenting with mild to moderate depression in primary care. The study involves 30 primary health centers in three districts in northern Uganda that were randomly assigned (with a 1:1 ratio) to have their LHWs trained in the delivery of GSP (intervention) or group HIV education (control condition) to PLWH with mild to moderate depression. The PLWH treated by the trained LHWs were evaluated at baseline, at the end of intervention, and at intervals of 6 months thereafter for 2 years.

**Figure 1:**
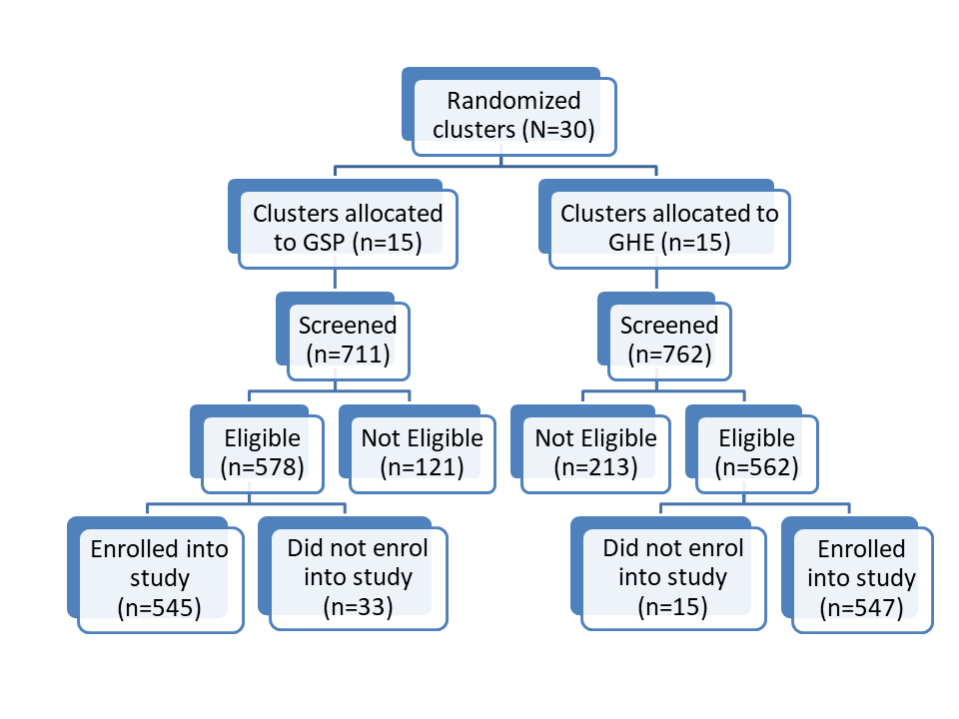
Recruitment process. GHE: group HIV education; GSP: group support psychotherapy.

The development of the GSP and group HIV education interventions has been described in detail in previous publications [19,21]. A detailed description of the content of both interventions has also been previously published [24]. The study was submitted to and approved by the Makerere University College of Health Sciences Research Ethics Committee, The AIDS Support Organization Research Ethics Committee, and the Uganda National Council of Science and Technology. All study participants were required to provide written informed consent. Light refreshments were served during all group sessions in both arms, and each participant and group facilitator received a financial incentive amounting to 8000UGX (US $2.16) and 80,000UGX (US $21.62), respectively, at the end of treatment to defray transportation costs. Figure 1 summarizes the trial profile.

### Training of Health Workers

#### Group Support Psychotherapy Training

Over a 4-month period (January to April 2016), Makerere University, in collaboration with the Ministry of Health, designed a GSP training program that consisted of both formal and informal training. This training was delivered using a training-of-trainers model in May and August 2016. Mental health specialists trained primary health center workers who, in turn, trained the LHWs. Formal training consisted of eight training modules delivered in a 5-day training workshop that employed active learning techniques including role plays, brainstorming sessions, and small group discussions. In brief, the first three modules included an overview of the training program, introduction to the GSP model, and an introduction to depression and HIV/AIDS were delivered on the first day. On the second and third days, modules on basic counseling skills and effective coping strategies were delivered, respectively. On the fourth day, participants received training in basic livelihood skills (enterprise selection, basic financial skills, and resource mobilization) required to overcome poverty. The last day of training focused on self-care strategies, posttraining assessments, and training workshop evaluation. Informal training consisted of conducting supervised pilot GSP sessions. Newly trained health workers were supervised by a pool of previously trained health workers who participated in the pilot randomized controlled trial [21]. The competencies targeted by the training have been published elsewhere [24]. Multimedia Appendix 1 shows the health worker supervision checklist used by supervisors.

#### Group HIV Education Training

In May 2016, Makerere University, in collaboration with The AIDS Support Organization, designed a group HIV education training program that consisted of both formal and informal training. Between May and August 2016, the training-of-trainers model was used to deliver the training, whereby The AIDS Support Organization HIV care providers trained primary health center workers who in turn trained the LHWs. Formal training consisted of five training modules delivered in a lecture format in a 2-day training workshop. In brief, on the first day, three modules including an overview of the training program, introduction to depression and HIV/AIDS, HIV progression, and transmission were delivered. On the second day, modules on mother-to-child transmission and basic facts on ART were delivered. Informal training consisted of conducting supervised pilot group HIV education sessions.

After both trainings, the trained LHWs initiated health education talks on depression in villages where they work and also among PLWH returning for medication refill. The PLWH who identified they had depression symptoms were invited for further evaluation. Those who met diagnostic criteria for mild to moderate depression were invited to attend group sessions of either GSP or group HIV education.

### Study Screening and Recruitment

We used study teams that reflected the ethnicity of the target community at each of the participating primary health centers. The study teams worked with the trained LHWs, who are the first level of health care delivery in the country, to spread information about the study by word of mouth in villages within the study region. The LHWs are members of the village health team [25]. They know individuals in the community who are receiving HIV care and could approach them directly with information about the study.

The study team conducted presentations in the community to explain study purpose and procedures to facilitate community understanding of the trial activities. At each participating primary health center, trained LHWs gave health talks on depression to clients in the waiting area. Those who had experienced symptoms of depression described in the health talk were invited for further evaluations using the Luo version of the 20-item Self-Reporting Questionnaire (SRQ-20) and the Mini International Neuropsychiatric Interview (MINI) depression module [26].

This procedure was repeated until a total of 40 PLWH meeting the MINI criteria of major depression were obtained from each primary health center. Research assistants approached each eligible client and explained study procedures, determined that other eligibility criteria were met and then obtained informed consent. To be recruited in the study, participants had to be HIV positive, aged 19 years or older, meet MINI criteria for major depressive disorder, antidepressant naïve, using ART, and residing in the villages where the trained LHWs lived. Individuals at high suicide risk [27], with a severe medical condition such as pneumonia or active tuberculosis, those with psychotic symptoms, or hearing or visual impairment were excluded from the study. Recruited participants from the same village were assigned to a trained LHW residing in or near their village to receive the intervention they had been trained to deliver (ie, either GSP or group HIV education).

### Study Measures

A structured questionnaire administered in the local language was used to collect data on a number of baseline variables in one-on-one, face-to-face interviews.

#### Baseline Sociodemographic Variables

Sociodemographic variables were assessed using a demographic questionnaire that asked about descriptive information including age, gender, number of children, education, and relationship and employment status. Employment status was categorized into ‘‘unemployed,” “employed,” and “peasant farmer.” Relationship status was categorized into ‘‘never married,” “married/living with a partner,” “divorced/ separated,” or “widowed.” Education status was categorized into “primary/no formal education” and “secondary and above.”

#### Baseline Psychiatric and Psychosocial Variables

##### Major Depressive Disorder

Major depressive disorder was diagnosed with the MINI depression module. The MINI is a diagnostic structured interview that was developed for the *Diagnostic and Statistical Manual of Mental Disorders* (4th Edition) of psychiatric disorders [26]. The psychometric properties of the MINI have not been described in Uganda; however, its depression diagnostic section has been translated and locally adapted in Luganda and previously used in this setting [28]. The depression diagnostic section consists of two screening questions, seven additional questions related to depression symptoms, and one question related to functional impairment. The two screening questions ask about the presence of depressed mood and loss of interest in daily activities over a period of 4 weeks in the recent past. If either one or both questions were positively endorsed by a study participant, the clinician asked additional questions to explore current (ie, 4 weeks before the interview) major depressive disorder. A diagnosis of current major depressive disorder was made if a study participant positively endorsed ﬁve or more questions related to depression symptoms and the one question related to functional impairment over a 4-week period.

##### Depression Symptoms

Depression symptoms were assessed using the SRQ-20 [29]. Cross-cultural adaptation and validation of the SRQ-20 in PLWH in southern Uganda showed that an optimum cut-off point of six or higher had a sensitivity of 84% and a specificity of 93% for current depression [30]. In this study sample, SRQ scores were modeled as a continuous variable with a Cronbach alpha reliability coefficient of .77.

##### Functioning Level

We assessed functioning levels using a five-item locally developed function assessment method [24]. Items were derived from qualitative interviews with individuals and their caregivers who were attending psychotrauma centers in Kitgum and Gulu [31].

In this population, the measure attained a Cronbach alpha of .86. The scale consisted of five categories of tasks including household (eg, washing clothes, sweeping the yard), work in the field (eg, digging, grazing animals), social interactions (eg, attending social events), and job-related or school-related tasks (eg, participating in income-generating activities, attending school or skills-training courses) and tasks related to personal hygiene (eg, bathing). Study participants were asked to indicate their ability to do a given task on a three-point scale where 0 referred to those who responded ‘‘no, I am not able,” 1 to ‘‘yes, but not like before,” and 2 to ‘‘yes, I am able to.”

##### Disability Days

We assessed disability days by asking a single question “How many working days have you lost due to depression-related symptoms in the previous 30 days?” Disability days reported were modeled as a continuous variable.

##### Posttraumatic Stress Symptoms

Posttraumatic stress symptoms were assessed using the locally adapted Harvard Trauma Questionnaire. It has been successfully translated into several languages, with acceptable measures of reliability and validity [32]. In this study population, the measure attained a Cronbach alpha reliability coefficient of .93.

##### Traumatic Experiences

War-related traumatic experiences were assessed using a locally developed 16-item trauma event checklist. Participants were asked whether they had experienced a given traumatic event or not. The trauma event checklist included items such as ‘‘Has the patient been forced to torture others?” “Has the patient witnessed torture/killing of another person?” “Has the patient been forced to kill?’’ A variable indicating the number of traumatic experiences by an individual was created and modeled as a continuous variable.

##### Perceived Social Support:

Perceived social support was assessed with the 12-item multidimensional social support scale [33]. The scale has been validated in Uganda and its three-subscale structure (family, friends, and significant other) was confirmed [34]. The Cronbach alpha for this sample was .96. Responses were based on a seven-point Likert scale with higher scores indicating greater support from friends, family, and significant others. We obtained total scores from the scale and modeled the scores as a continuous variable.

##### Self-Esteem

Self-esteem was measured using the 10-item Rosenberg Self-Esteem Scale, which provides assessment of one’s general feelings about oneself [35]. Responses were based on a four-point scale. This scale has been used in HIV-positive women in South Africa [36]. The scores range from 10 to 40 with higher scores indicating higher self-esteem. We obtained total scores from the scale and modeled the scores as a continuous variable. In this study sample, the measure attained a Cronbach alpha of .93.

##### Alcohol Use

We assessed alcohol use with the Alcohol Use Disorders Identification Test (AUDIT) [37]. This 10-item scale has been validated in PLWH populations in sub-Saharan Africa [10]. Each of the 10 questions is rated on a four-point scale. The total score ranges from 0 to 40. A total of eight or higher is recommended as an indicator of hazardous drinking behavior and a score of 20 or higher is indicative of alcohol dependence. We report this variable both as categorical using the cut-off point of eight or higher and continuous using total AUDIT scores. In this study population, the measure attained a Cronbach alpha of .95.

##### Coping Skills

We used the modified coping inventory to assess a broad range of both positive and negative coping responses which establish how the study participants responded when they were confronted with difficult or stressful events in their lives [38]. Each coping strategy is assessed by a set of two questions. Responses were based on a four-point scale. For each coping strategy, the scores range from two to eight, with higher scores indicating frequent use of the coping strategy. Each coping strategy was modeled as a continuous variable.

##### Stigma and Discrimination

To measure internalized stigma, we used the brief AIDS-related stigma scale [39]. Responses were based on a four-point Likert scale. The scores ranged from 8 to 32 with high scores indicating higher levels of internalized stigma.

To measure discrimination (enacted stigma), we used a total of 13 items adapted from an HIV/AIDS indicator survey previously used by Nyblade and MacQuarrie [40] which described various forms of discriminatory events experienced by PLWH as a result of their HIV status. Both stigma and discrimination were modeled as continuous variables.

##### Viral Load

Once a year, HIV treatment centers routinely assess the viral load of clients. Measures of viral load were obtained from the medical charts of study participants. Although the name of the assay used to measure viral load in the laboratory was not recorded, all viral load measures were conducted by the same laboratory. If the record in the chart noted undetectable viral copies, that individual was assumed to have achieved viral suppression. If there was a record of detected viral copies in the chart, these were transcribed to the study questionnaire. Viral load was treated both as a continuous variable and as a categorical variable indicating suppression (coded 1) or nonsuppression of viral load (coded 0).

##### Adherence to Antiretroviral Therapy

Adherence to ART was assessed by using the missed-dose method, which is simple to implement and straightforward. Participants were asked to report the number of missed doses within a specified time period. We asked a single question: “During the past week, on how many days have you missed taking all your medication doses?” Adherence was calculated as the percent of days in the week the person missed all medications. Adherence “scores” were treated both as a continuous variable in correlation analyses and also converted to a dichotomous variable (eg, adherent/nonadherent) to simplify their interpretation for the descriptive analyses. In previous studies, researchers have used either 80% or 90% or 100% as the cut-off point for adherence [41,42]. In this study, we used 100% as the cut-off point.

##### Socioeconomic Status Index

To evaluate the baseline socioeconomic status (SES) of study participants, we created an SES index using principal component analysis of the following variables: presence or absence of an income-generating activity, food security, savings, and household assets (land, animals, poultry, radio, television, mobile phone, bicycle, or motorcycle). The index was categorized into quintiles with the first quintile representing low SES, the second to fourth quintiles representing the medium SES, and the fifth quintile representing the high SES group.

### Statistical Analyses

Statistical analyses were performed using STATA statistical software version 15. Descriptive statistics were used to describe the demographic, clinical, and psychosocial characteristics of the study population. Among eligible participants, differences between those enrolled versus those who refused enrollment were assessed using chi square tests for categorical variables and *t* tests for continuous variables. Due to violations of normality, nonlinear relationships and the presence of ordinal variables, we opted to use the nonparametric Spearman rank order correlation (ρ coefficient) to examine associations between depression symptoms and other baseline variables. A Bonferroni adjustment was applied to calculated significance levels. Statistical significance was considered at two-tailed *P* ≤.05.

## Results

### Screening and Recruitment

The study recruitment period was from September to December 2016. After a series of health talks on depression in 30 participating primary health centers, a total of 1473 individuals expressed interest in being screened for depression. Of these, 1140 were eligible and enrolled in the study. On commencement of the interventions, 48 (4.21%) did not attend any group session. Of these, 21 had failed to complete baseline assessments, while 27 had completed baseline assessments. Although these participants appeared to have withdrawn from the study after enrollment, they did not announce their exit. Therefore, we still consider them as part of the study. Figure 1 summarizes the recruitment process.

The study was able to recruit 95% the target sample size of 1200 described in the study protocol. The target of 40 participants in each health center was met by only 14 primary health centers. Only 23 participants were enrolled outside the target enrollment period. Multimedia Appendices 2 and 3 show the geographical distribution of the primary health centers and the actual enrollment versus target enrollment, respectively.

Other protocol deviations reported included the enrollment of 141 of 1140 (12.37%) individuals who did not meet the MINI criteria for major depression. However, of those enrolled, 127 of 1140 (11.14%) were judged to be clinically depressed by the health workers and had attained a score of more than six on the SRQ-20, which indicates a high probability of major depression [30]. In addition, 13 of 1140 (1.14%) did not meet the criteria for depression and 22 of 1140 (2.02%) did not have a record of their depression status. Individuals with high suicide risk were to be excluded from the study; however, for 32 of 1140 (2.80%) individuals with high suicide risk, the health workers insisted that they be included in the study as the group sessions were the only interventions accessible at that time and it would be unethical if they did not receive any form of intervention for their depression. Multimedia Appendix 4 illustrates details of the protocol deviations.

### Baseline Characteristics of Study Participants

The sample consisted of slightly more women (612/1140, 53.68%) than men (528/1140, 46.32%). The age of participants ranged from 19 to 80 years, with a mean age of 38.5 (SD 10.9) years. The majority were peasant farmers (622/1140, 54.56%), with primary level education or less (983/1140, 86.22%), without an income-generating activity (828/1140, 72.63%), and without food security (931/1140, 81.67%). Most participants met criteria for major depression (1105/1140, 96.92%) at baseline, as described previously, and reported moderate suicide risk (599/1140, 52.54%). Participants’ function scores ranged from 0 to 10, with a mean function score of 4.98 (SD 2.91). In all, 72.46% of study participants (826/1140) met the criteria for probable posttraumatic stress disorder, and 27.46% (313/1140) met the criteria for hazardous alcohol consumption. Tables 1 and 2 provide detailed descriptions of participant baseline sociodemographic and psychosocial characteristics, respectively.

Participants who did not attend any group sessions were significantly more likely to be male than females (χ^2^_1_=4.0, *P*=.045) and on average had more depression symptoms (*t*_*2*_=2.36, *P*=.01) than participants who attended group sessions. Table 3 provides detailed comparisons of study participants who attended versus those who did not attend any group sessions.

**Table 1:** Baseline sociodemographic characteristics of the study participants (N=1140).

Variable		Participants
**Age (years)**		
	Mean (SD)	38.5 (10.9)
	Range	19-80
**Gender, n (%)**		
	Female	612 (53.68)
	Male	528 (46.32)
**Educational background, n (%)**		
	Primary education or lower	983 (86.23)
	Secondary education or higher	157 (13.77)
**Employment status, n (%)**		
	Not employed	405 (35.53)
	Employed	113 (9.91)
	Peasant farmer	622 (54.56)
**Relationship status, n (%)**		
	Never married	151 (13.25)
	Married or living with partner	816 (71.58)
	Separated or divorced	87 (7.63)
	Widowed	86 (754)
**Has income-generating activity, n (%)**		
	Yes	312 (22.37)
	No	828 (72.63)
**Has savings, n (%)**		
	Yes	302 (26.49)
	No	838 (73.51)
**Has a leadership position in the community, n (%)**		
	Yes	171 (15.00)
	No	648 (85.00)
**Has food security, n (%)**		
	Yes	209 (18.33)
	No	931 (81.67)
**Number of children**		
	Mean (SD)	4.0 (2.0)
	Range	0-16
**Disability days in the past month**		
	Mean (SD)	8.5 (9.2)
	Range	0-30
**Baseline monthly income (UGX)**		
	Mean (SD)	7476 (17,651)
	Range	0-150,000
**Baseline savings(UGX)**		
	Mean (SD)	13,758 (40,964)
	Range	0-400,000
**Social economic status index**		
	Low (quintile 1)	342 (38.78)
	Medium (quintiles 2-4)	485 (54.99)
	High (quintile 5)	55 (6.24)
	Missing	258 (22.63)

**Table 2:** Baseline psychosocial characteristics of the study participants (N=1140).

Variable		Participants
**Major depression, n (%)**		
	Met MINI criteria	978 (85.78)
	Clinically depressed	127 (11.14)
	Did not meet any criteria	13 (1.14)
	Missing data	22 (1.93)
**Suicide risk, n (%)**		
	Low	479 (42.02)
	Moderate	599 (52.54)
	High	32 (2.80)
	Missing	30 (2.63)
Depression score, mean (SD); range		13.6 (4); 0-20
Posttraumatic stress score, mean (SD); range		42.6 (12.5); 16-64
Function score, mean (SD); range		5 (2.9); 0-10
Social support score, mean (SD); range		44 (20); 0-84
Self-esteem score, mean (SD); range		14 (8); 0-32
Internalized stigma score, mean (SD); range		22 (5); 8-32
Alcohol Use(AUDIT) score, mean (SD); range		6 (9.0); 0-40
**Positive coping skills, mean (SD); range**		
	Self-distraction	3.7 (2.0); 2-8
	Active coping	4.2 (2.0); 1-8
	Use of emotional support	4 (2); 2.0-8
	Use of venting	3.9 (2.0); 2-8
	Acceptance	4.2 (2.0); 1-8
	Use of positive reframing	4 (2.0); 1-8
	Use of religion	4.7 (2.0); 0-8
**Negative coping skills, mean (SD); range**		
	Denial	5 (2.0); 0-8
	Self-blame	2.8 (1.2); 0-4
	Use of alcohol and substances	4.55 (2.6); 0-8
	Behavioral disengagement	5.2 (2.0); 0-8
**Enacted stigma (discrimination), mean (SD); range**		
	Isolation	2.8 (3.0); 0-10
	Verbal abuse	2.2 (1.4); 0-4
	Loss of identity	1.2 (1.2); 0-4
	Unable to access community resources	2.6 (3.0); 0-10

**Table 3:** A comparison of eligible study participants who attended versus those who did not attend any group sessions (N=1140).

Variable		Attended (n=1092)	Did not attend (n=48)	Effect estimate		*P* value
				Χ^2^_1_	*t*_2_	
Age (years), mean (SD)		38.5 (10.9)	37 (8.7)		0.81	.20
**Gender, n (%)**				4.0		.04
	Female	593 (54.30)	19 (39.6)			
	Male	499 (45.69)	29 (60.4)			
**Educational background, n (%)**				1.1		.31
	Primary education or lower	944 (86.45)	39 (81.3)			
	Secondary education or higher	148 (13.55)	9 (18.75)			
**Occupational status, n (%)**				1.3		.53
	Not employed	385 (35.25)	20 (41.7)			
	Employed	110 (10.10)	3 (6.3)			
	Peasant farmer	597 (54.67)	25 (52.1)			
**Relationship status, n (%)**				3.9		.27
	Never married	149 (13.65)	2 (4.2)			
	Married or living with partner	779 (71.16)	37 (77.1)			
	Separated or divorced	82 (7.51)	5 (10.4)			
	Widowed	82 (7.51)	4 (8.3)			
Depression score, mean (SD)		13.6 (2.4)	11.9 (4.4)		2.36	.01

### Correlations Among Depression and Related Psychosocial Variables

There were several signiﬁcant associations found between depression symptoms and both clinical and psychosocial variables at baseline. Depression symptoms assessed by the MINI were positively correlated with posttraumatic stress symptoms (ρ=.43, *P*<.001), suicide risk (ρ=.32, *P*<.001), stigma (ρ=.30, *P*<.001), number of traumatic experiences (ρ=.25, *P*<.001), and negatively correlated with perceived social support (ρ=–.35, *P*<.001), functioning (ρ=–.22, *P*<.001), self-esteem (ρ=–.39, *P*<.001), and adherence to ART(ρ=–.12, *P*=.05). Suicide risk was positively correlated with posttraumatic stress symptoms (ρ=.28, *P*<.001), number of traumatic experiences (ρ=.21, *P*<.001), stigma (ρ=.2, *P*<.001), and alcohol use (ρ=.21, *P*<.001).

Adherence to ART was positively correlated with social support (ρ=.15, *P*<.001) and negatively correlated with stigma (ρ=–.14, *P*<.001). The number of traumatic experiences was positively correlated with viral load (ρ=.12, *P*=.05). Correlations between viral load and depression symptoms were positive but weak and nonsignificant. See Table 4 for the correlation matrix.

**Table 4:** Baseline correlations among depression symptoms and related psychosocial variables (N=1140). *P<.001; **P<.05.

Symptoms and variables	Depression	Suicide risk	Function	PTSD^a^	Social support	Self-esteem	Stigma	Viral load	Adherence	Trauma	Alcohol Use	SES^b^ Index
Depression												
Suicide risk	.32*											
Function	–.22*	–.17*										
PTSD	.43*	.28*	–.36*									
Social support	–.35*	–.22*	.28*	–.33*								
Self-esteem	–.40*	–.21*	.51*	–.48*	.46*							
Stigma	.30*	.20*	–.26*	.28*	–.33*	–.42*						
Viral load	.02	.05	.07	–.02	.04	.08	–.06					
Adherence	–.11**	–.01	–.00	–.04	.15*	.06	–.14*	.05				
Trauma	.24*	.21*	–.11**	.13*	–.06	–.08	.05	.12**	.05			
Alcohol use	–.08	.22*	.04	–.06	–.01	.09	.06	–.05	.01	.13*	1.00	
SES Index	–.04	.15*	.02	–.03	.09	.07	–.02	.03	–.01	.02	0.02	1.00

^a^PTDS: posttraumatic stress disorder.

^a^SES: socioeconomic status.

## Discussion

The SEEK-GSP study tests GSP delivered by trained LHW for treatment of depression in rural areas in postconflict northern Uganda. The recruitment goal for the study was largely achieved, with 95% of the targeted sample size enrolled in the trial in a 14-week period. This finding suggests that participants were easy to recruit for the study and were willing to participate in either study arm. In contrast to previous trials of psychological interventions for depression in sub-Saharan Africa [43-45], 80% of those recruited in the study attended all group sessions. This high attendance, which was also observed in our previous pilot trial [24], indicates keen interest and confirms the acceptability of group interventions in the target population.

Among trials of psychological interventions to treat depression in PLWH in sub-Saharan Africa, this study is the ﬁrst to enroll a large sample of males. The majority of previous trials of psychological interventions for depression have largely focused on women, including studies by Kaaya and colleagues from Tanzania [44], Chibanda and colleagues from Zimbabwe [45], and Bolton and colleagues from southern Uganda [46]. These studies are important given the dearth of prior studies focused on psychological treatments for depression in sub-Saharan Africa. However, interventions that attract men may be particularly relevant to African communities and may be a promising avenue for engaging them in research and, if eﬃcacious, improving the health of the entire community.

The baseline data from this study indicate that PLWH with depression are vulnerable on multiple levels and disadvantaged across many social and economic determinants of health. The majority have low education, lack an income-generating activity and food security, and on average were unable to work for 10 days in the month prior to enrollment. These findings are not surprising. Such socioeconomic disadvantage has been documented among HIV-positive populations across the African continent [47,48] and is often associated with depression [49,50].

Correlation analyses indicate that there are significant positive and negative correlations between depression symptoms and other baseline variables. The positive correlations between depression and posttraumatic symptoms, internalizing stigma and number of traumatic experiences, are not surprising. Several studies have demonstrated the comorbidity of stigma, depression, and posttraumatic stress symptoms in primary care settings [51-53].

Among the negative correlations, a larger number of depression symptoms were associated with lower levels of functioning, perceived social support, and self-esteem. Previous studies have confirmed that depression impairs functioning [54,55], while social support is the most potent buffer against depression [56-58]. Further, a meta-analysis of 77 longitudinal studies provides consistent support for the fact that low self-esteem contributes to depression [5].

Our study findings further indicate that with a larger number of depression symptoms, there is a lesser degree of adherence to ART, confirming what a myriad of previous studies have shown worldwide [59]. We also found a significant positive correlation between the number of traumatic experiences and viral load. Prior longitudinal studies in high-income countries have found a similar association [60,61]. However, such an association has not been documented among PLWH in sub-Saharan Africa. Overall, our study findings justify the active ingredients of our experimental intervention—GSP which places emphasis on enhancing emotional and social support, coping skills to combat stigma and discrimination, and support economic empowerment.

This study has a number of limitations. Although our data are drawn from multiple HIV clinics across three districts in northern Uganda, the study participants share the same ethnicity. Given that Uganda is a multiethnic country, our results may not be applicable to other ethnic populations. The study needs to be replicated in other regions of the country. Self-report is not the most reliable measure of ART adherence; therefore, our adherence estimates may represent an overestimate. However, a gold standard for adherence assessment does not exist and different assessment methods have been used in different studies [62].

Despite these limitations, the SEEK-GSP study provides a rich dataset with follow-up of the high number of persons with major depressive disorder and HIV disease in sub-Saharan Africa. The large sample size will allow us to conduct subgroup analyses to assess how some variables, such as gender and psychiatric comorbidities (hazardous alcohol consumption and posttraumatic stress), can modify the effects of GSP on depression. Identifying groups of individuals for whom GSP works best will assist future work toward developing selection criteria to guide referral of patients for GSP. Additionally, variables that mediate the effects of GSP on depression, and subsequently other study outcomes, can thus be identified.

## Appendix

Multimedia Appendix 1 Health Worker Supervision Checklist.

Multimedia Appendix 2 Geographical distribution of primary health care centers in the study.

Multimedia Appendix 3 Actual versus expected enrolment of study participants.

Multimedia Appendix 4 Study protocol deviations.

## Abbreviations

ART: antiretroviral therapy
AUDIT: Alcohol Use Disorders Identification Test
GSP: group support psychotherapy
LHW: lay health worker
MINI: Mini International Neuropsychiatric Interview
PLWH: person living with HIV
SEEK-GSP: Social, Emotional, and Economic Empowerment Through Knowledge of Group Support Psychotherapy
WHO: World Health Organization

## References

[1] Nakimuli-Mpungu E, Bass JK, Alexandre P, Mills EJ, Musisi S, Ram M, Katabira E, Nachega JB (2012). Depression, alcohol use and adherence to antiretroviral therapy in sub-Saharan Africa: a systematic review. AIDS Behav.

[2] Bernard C, Dabis F, de Rekeneire N (2017). Prevalence and factors associated with depression in people living with HIV in sub-Saharan Africa: A systematic review and meta-analysis. PLoS One.

[3] Li L, Lin C, Liang L, Ji G (2016). Exploring coping and social support with gender and education among people living with HIV in China. AIDS Behav.

[4] Yeji F, Klipstein-Grobusch K, Newell M, Hirschhorn LR, Hosegood V, Bärnighausen T (2014). Are social support and HIV coping strategies associated with lower depression in adults on antiretroviral treatment? Evidence from rural KwaZulu-Natal, South Africa. AIDS Care.

[5] Sowislo JF, Orth U (2013). Does low self-esteem predict depression and anxiety? A meta-analysis of longitudinal studies. Psychol Bull.

[6] Familiar I, Murray S, Ruisenor-Escudero H, Sikorskii A, Nakasujja N, Boivin MJ, Opoka R, Bass JK (2016). Socio-demographic correlates of depression and anxiety among female caregivers living with HIV in rural Uganda. AIDS Care.

[7] Mahajan AP, Sayles JN, Patel VA, Remien RH, Sawires SR, Ortiz DJ, Szekeres G, Coates TJ (2008). Stigma in the HIV/AIDS epidemic: a review of the literature and recommendations for the way forward. AIDS.

[8] Lowther K, Selman L, Harding R, Higginson IJ (2014). Experience of persistent psychological symptoms and perceived stigma among people with HIV on antiretroviral therapy (ART): a systematic review. Int J Nurs Stud.

[9] Olley BO, Zeier MD, Seedat S, Stein DJ (2005). Post-traumatic stress disorder among recently diagnosed patients with HIV/AIDS in South Africa. AIDS Care.

[10] Myer L, Smit J, Roux LL, Parker S, Stein DJ, Seedat S (2008). Common mental disorders among HIV-infected individuals in South Africa: prevalence, predictors, and validation of brief psychiatric rating scales. AIDS Patient Care STDS.

[11] Boarts JM, Sledjeski EM, Bogart LM, Delahanty DL (2006). The differential impact of PTSD and depression on HIV disease markers and adherence to HAART in people living with HIV. AIDS Behav.

[12] Katz IT, Ryu AE, Onuegbu AG, Psaros C, Weiser SD, Bangsberg DR, Tsai AC (2013). Impact of HIV-related stigma on treatment adherence: systematic review and meta-synthesis. J Int AIDS Soc.

[13] Ironson G, O'Cleirigh C, Kumar M, Kaplan L, Balbin E, Kelsch CB, Fletcher MA, Schneiderman N (2015). Psychosocial and neurohormonal predictors of HIV disease progression (CD4 cells and viral load): a 4 year prospective study. AIDS Behav.

[14] Delpierre C, Cuzin L, Lauwers-Cances V, Datta GD, Berkman L, Lang T (2008). Unemployment as a risk factor for AIDS and death for HIV-infected patients in the era of highly active antiretroviral therapy. Sex Transm Infect.

[15] Aibibula W, Cox J, Hamelin A, McLinden T, Klein MB, Brassard P (2017). Association between food insecurity and HIV viral suppression: a systematic review and meta-analysis. AIDS Behav.

[16] van der Heijden I, Abrahams N, Sinclair D (2017). Psychosocial group interventions to improve psychological well-being in adults living with HIV. Cochrane Database Syst Rev.

[17] Chibanda D, Cowan FM, Healy JL, Abas M, Lund C (2015). Psychological interventions for common mental disorders for people living with HIV in low- and middle-income countries: systematic review. Trop Med Int Health.

[18] Murry VM, Brown PA, Brody GH, Cutrona CE, Simons RL (2001). Racial discrimination as a moderator of the links among stress, maternal psychological functioning, and family relationships. J Marriage and Family.

[19] Nakimuli-Mpungu E, Wamala K, Okello J, Alderman S, Odokonyero R, Musisi S, Mojtabai R (2014). Developing a culturally sensitive group support intervention for depression among HIV infected and non-infected Ugandan adults: a qualitative study. J Affect Disord.

[20] Nakimuli-Mpungu E, Wamala K, Okello J, Alderman S, Odokonyero R, Musisi S, Mojtabai R, Mills EJ (2014). Outcomes, feasibility and acceptability of a group support psychotherapeutic intervention for depressed HIV-affected Ugandan adults: a pilot study. J Affect Disord.

[21] Nakimuli-Mpungu E, Wamala K, Okello J, Alderman S, Odokonyero R, Mojtabai R, Mills EJ, Kanters S, Nachega JB, Musisi S (2015). Group support psychotherapy for depression treatment in people with HIV/AIDS in northern Uganda: a single-centre randomised controlled trial. The Lancet HIV.

[22] Roberts B, Ocaka KF, Browne J, Oyok T, Sondorp E (2008). Factors associated with post-traumatic stress disorder and depression amongst internally displaced persons in northern Uganda. BMC Psychiatry.

[23] Nakimuli-Mpungu E, Musisi S, Wamala K, Okello J, Ndyanabangi S, Mojtabai R, Nachega J, Harari O, Mills E (2017). The effect of group support psychotherapy delivered by trained lay health workers for depression treatment among people with HIV in Uganda: protocol of a pragmatic, cluster randomized trial. JMIR Res Protoc.

[24] Nakimuli-Mpungu E, Wamala K, Okello J, Ndyanabangi S, Kanters S, Mojtabai R, Nachega JB, Mills EJ, Musisi S (2017). Process evaluation of a randomized controlled trial of group support psychotherapy for depression treatment among people with HIV/AIDS in Northern Uganda. Community Ment Health J.

[25] Baingana F, Mangen PO (2011). Scaling up of mental health and trauma support among war affected communities in northern Uganda. Intervention.

[26] Sheehan DV, Lecrubier Y, Sheehan KH, Amorim P, Janavs J, Weiller E, Hergueta T, Baker R, Dunbar GC (1998). The Mini-International Neuropsychiatric Interview (M.I.N.I.): the development and validation of a structured diagnostic psychiatric interview for DSM-IV and ICD-10. J Clin Psychiatry.

[27] Patterson WM, Dohn HH, Bird J, Patterson GA (1983). Evaluation of suicidal patients: The SAD PERSONS scale. Psychosomatics.

[28] Muhwezi WW, Okello ES, Neema S, Musisi S (2008). Caregivers' experiences with major depression concealed by physical illness in patients recruited from central Ugandan Primary Health Care Centers. Qual Health Res.

[29] Beusenberg M, Orley J, World Health Organization (1994). A User's Guide to the Self-Reporting Questionnaire (SRQ).

[30] Nakimuli-Mpungu E, Mojtabai R, Alexandre PK, Katabira E, Musisi S, Nachega JB, Bass JK (2012). Cross-cultural adaptation and validation of the self-reporting questionnaire among HIV+ individuals in a rural ART program in southern Uganda. HIV AIDS (Auckl).

[31] Nakimuli-Mpungu E, Alderman S, Kinyanda E, Allden K, Betancourt TS, Alderman JS, Pavia A, Okello J, Nakku J, Adaku A, Musisi S (2013). Implementation and scale-up of psycho-trauma centers in a post-conflict area: a case study of a private-public partnership in northern Uganda. PLoS Med.

[32] Mollica R (2004). Harvard Program in Refugee Trauma.

[33] Zimet GD, Dahlem NW, Zimet SG, Farley GK (1988). The Multidimensional Scale of Perceived Social Support. J Pers Assess.

[34] Nakigudde J, Musisi S, Ehnvall A, Airaksinen E, Agren H (2009). Adaptation of the multidimensional scale of perceived social support in a Ugandan setting. Afr Health Sci.

[35] Rosenberg M (1965). Society and the Adolescent Self-Image.

[36] Mundell JP, Visser MJ, Makin JD, Kershaw TS, Forsyth BW, Jeffery B, Sikkema KJ (2011). The impact of structured support groups for pregnant South African women recently diagnosed HIV positive. Women Health.

[37] Nakamura Akinobu, Osonoi Takeshi, Terauchi Yasuo (2010). Relationship between urinary sodium excretion and pioglitazone-induced edema. J Diabetes Investig.

[38] Carver C, Scheier M, Weintraub J (1989). Assessing coping strategies: a theoretically based approach. J Pers Soc Psychol.

[39] Kalichman SC, Simbayi LC, Jooste S, Toefy Y, Cain D, Cherry C, Kagee A (2005). Development of a brief scale to measure AIDS-related stigma in South Africa. AIDS Behav.

[40] Nyblade L, MacQuarrie K (2017). Can We Measure HIV/AIDS-Related Stigma and Discrimination? Current Knowledge About Quantifying Stigma in Developing Countries.

[41] Liu H, Golin CE, Miller LG, Hays RD, Beck CK, Sanandaji S, Christian J, Maldonado T, Duran D, Kaplan AH, Wenger NS (2001). A comparison study of multiple measures of adherence to HIV protease inhibitors. Ann Intern Med.

[42] Golin CE, Liu H, Hays RD, Miller LG, Beck CK, Ickovics J, Kaplan AH, Wenger NS (2002). A prospective study of predictors of adherence to combination antiretroviral medication. J Gen Intern Med.

[43] Petersen I, Hanass Hancock J, Bhana A, Govender K (2014). A group-based counselling intervention for depression comorbid with HIV/AIDS using a task shifting approach in South Africa: a randomized controlled pilot study. J Affect Disord.

[44] Kaaya SF, Blander J, Antelman G, Cyprian F, Emmons KM, Matsumoto K, Chopyak E, Levine M, Smith Fawzi MC (2013). Randomized controlled trial evaluating the effect of an interactive group counseling intervention for HIV-positive women on prenatal depression and disclosure of HIV status. AIDS Care.

[45] Chibanda D, Weiss HA, Verhey R, Simms V, Munjoma R, Rusakaniko S, Chingono A, Munetsi E, Bere T, Manda E, Abas M, Araya R (2016). Effect of a primary care-based psychological intervention on symptoms of common mental disorders in Zimbabwe: a randomized clinical trial. JAMA.

[46] Bolton P, Bass J, Neugebauer R, Verdeli H, Clougherty KF, Wickramaratne P, Speelman L, Ndogoni L, Weissman M (2003). Group interpersonal psychotherapy for depression in rural Uganda: a randomized controlled trial. JAMA.

[47] Buvé A, Bishikwabo-Nsarhaza K, Mutangadura G (2002). The spread and effect of HIV-1 infection in sub-Saharan Africa. Lancet.

[48] Wojcicki JM (2005). Socioeconomic status as a risk factor for HIV infection in women in East, Central and Southern Africa: a systematic review. J Biosoc Sci.

[49] Lund C, Breen A, Flisher AJ, Kakuma R, Corrigall J, Joska JA, Swartz L, Patel V (2010). Poverty and common mental disorders in low and middle income countries: a systematic review. Soc Sci Med.

[50] Richardson R, Westley T, Gariépy G, Austin N, Nandi A (2015). Neighborhood socioeconomic conditions and depression: a systematic review and meta-analysis. Soc Psychiatry Psychiatr Epidemiol.

[51] Bonfils KA, Lysaker PH, Yanos PT, Siegel A, Leonhardt BL, James AV, Brustuen B, Luedtke B, Davis LW (2018). Self-stigma in PTSD: prevalence and correlates. Psychiatry Res.

[52] Greene T, Neria Y, Gross R (2016). Prevalence, detection and correlates of PTSD in the primary care setting: a systematic review. J Clin Psychol Med Settings.

[53] Hernandez D, Kalichman SC, Katner HP, Burnham K, Kalichman MO, Hill M (2018). Psychosocial complications of HIV/AIDS-metabolic disorder comorbidities among patients in a rural area of southeastern United States. J Behav Med.

[54] Rock PL, Roiser JP, Riedel WJ, Blackwell AD (2014). Cognitive impairment in depression: a systematic review and meta-analysis. Psychol Med.

[55] Fried EI, Nesse RM (2014). The impact of individual depressive symptoms on impairment of psychosocial functioning. PLoS One.

[56] Cobb S (1976). Presidential address-1976: social support as a moderator of life stress. Psychosom Med.

[57] Cohen S, Wills TA (1985). Stress, social support, and the buffering hypothesis. Psychol Bull.

[58] Stein ER, Smith BW (2015). Social support attenuates the harmful effects of stress in healthy adult women. Soc Sci Med.

[59] Uthman OA, Magidson JF, Safren SA, Nachega JB (2014). Depression and adherence to antiretroviral therapy in low-, middle- and high-income countries: a systematic review and meta-analysis. Curr HIV/AIDS Rep.

[60] Mugavero MJ, Pence BW, Whetten K, Leserman J, Swartz M, Stangl D, Thielman NM (2007). Predictors of AIDS-related morbidity and mortality in a southern US cohort. AIDS Patient Care STDS.

[61] Ironson G, O'Cleirigh C, Fletcher MA, Laurenceau JP, Balbin E, Klimas N, Schneiderman N, Solomon G (2005). Psychosocial factors predict CD4 and viral load change in men and women with human immunodeficiency virus in the era of highly active antiretroviral treatment. Psychosom Med.

[62] Ammassari A, Trotta MP, Murri R, Castelli F, Narciso P, Noto P, Vecchiet J, D'Arminio MA, Wu AW, Antinori A, AdICoNA Study Group (2002). Correlates and predictors of adherence to highly active antiretroviral therapy: overview of published literature. J Acquir Immune Defic Syndr.

