# Supplementary material for: Recruitment and Baseline Characteristics of Participants in the Social, Emotional, and Economic Empowerment Through Knowledge of Group Support Psychotherapy Study (SEEK-GSP): Cluster Randomized Controlled Trial
Source: JMIR Res Protoc. 2019 Jan 3;8(1):e11560. doi: 10.2196/11560 (PMC6682267; doi:10.2196/11560)
Supplement: Multimedia Appendix 1 [file resprot_v8i1e11560_app1.pdf]

SUPERVISION FORM

STUDY ID OF TRAINEE………………………………………………… QUALIFICATION……………………………………………………

SUPERVISOR ID/ NAME…………………………………………… QUALIFICATION……………………………………………………

HEALTH CENTER………

GROUP SUPPORT PSYCHOTHERAPY SESSION TITLE and NO: ………………………………………………………………………

Health Worker Supervision Checklist. Please refer to the supervisor's guide then tick the appropriate box
If a particular skill is not applicable to the group session indicate N/A

**Counseling skills**	**Needs improvement**	**Done partially**	**Done well**
1. The health worker is able to obtain and address group members expectations from the group session			
2. The health worker demonstrates appropriate facial expression, eye contact, gestures &body language			
3. The health worker asks appropriate questions, Uses summarizing and clarifying statements			
4. The health worker is able to build rapport & encourages group members to express themselves			
5. The health worker demonstrates ability to explore and normalize group members feelings			
6. The health worker is able to demonstrate EMPATHY, WARMTH & GENUINENESS			
7. The health worker is able to explores members own explanations regarding the cause of their problems			
8. The health worker is able to explore the group members social support networks			
9. The health worker is able to promote realistic hope for change			
10. The health worker is able to promote confidentiality in each group session			
11. The health worker is able to explore group members feelings & thoughts of self harm and harm to others			
12. The health worker is able to encourage feedback from group members regarding any advice given			
13. The health worker is able to explore the group members own coping mechanisms and advise on other effective ways of coping.			
14. The health worker is able to use the group support psychotherapy manual effectively			
