# Supplementary figures and images for: Recruitment and Baseline Characteristics of Participants in the Social, Emotional, and Economic Empowerment Through Knowledge of Group Support Psychotherapy Study (SEEK-GSP): Cluster Randomized Controlled Trial
Source: JMIR Res Protoc. 2019 Jan 3;8(1):e11560. doi: 10.2196/11560 (PMC6682267; doi:10.2196/11560)

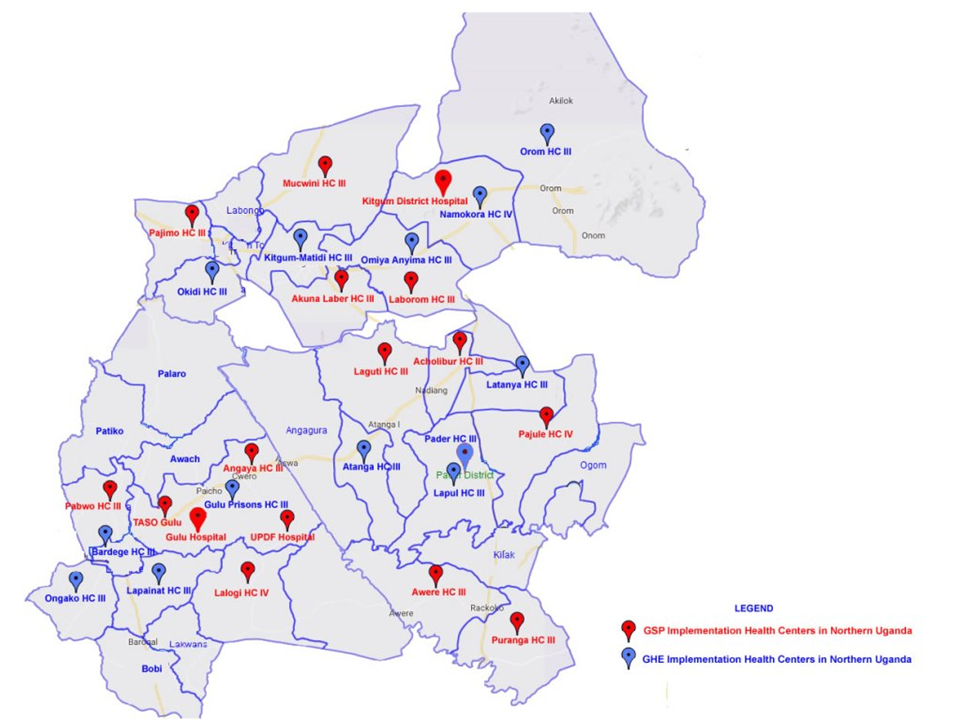

Supplement: Multimedia Appendix 2 [file resprot_v8i1e11560_app2.png]

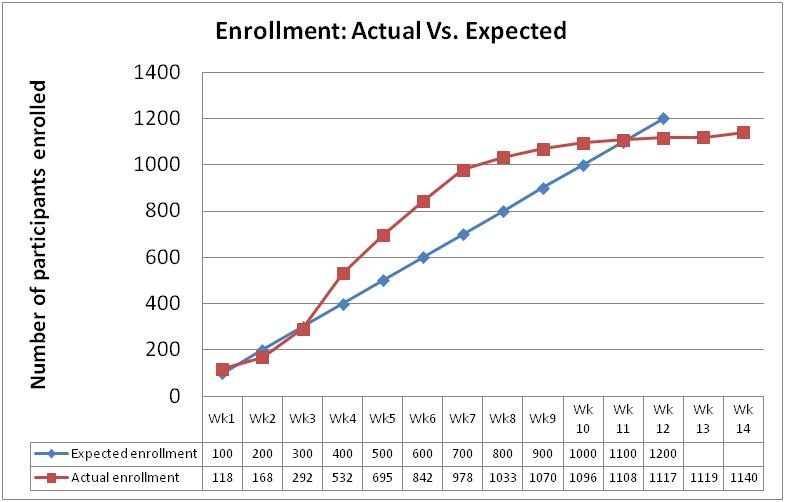

Supplement: Multimedia Appendix 3 [file resprot_v8i1e11560_app3.png]
