# Supplementary material for: Recruitment and Baseline Characteristics of Participants in the Social, Emotional, and Economic Empowerment Through Knowledge of Group Support Psychotherapy Study (SEEK-GSP): Cluster Randomized Controlled Trial
Source: JMIR Res Protoc. 2019 Jan 3;8(1):e11560. doi: 10.2196/11560 (PMC6682267; doi:10.2196/11560)
Supplement: Multimedia Appendix 4 [file resprot_v8i1e11560_app4.pdf]

PROTOCOL DEVIATIONS

Table 11: Off-Protocol Events by Site

**Off-Protocol Events**	Gulu (N=379) N(%)	Kitgum (N=375) N(%)	Pader (N=386) N(%)	Total (N=1140)
Participants enrolled outside the target period	8(2.11)	10(2.67)	5(1.30)	23(2.02)
Number of health centers that did not meet the target enrolment **(N=30)**	8(80)	5(50)	3(30)	16(53.33)
Enrolled participants did not meet criteria for major depression	1(7.69)	7(53.85)	5(38.46)	13(1.14)
Enrolled participants with high suicide risk	4(12.50)	5(15.63)	23(71.88)	32(2.81)
Did not have baseline assessments	12(57.14)	4(19.05)	5(23.81)	21(1.84)
Lost to follow up after end of treatment	37(57.81)	11(17.19)	16(25)	64(5.61)
**Group session attendance of trial participants**				
Did not attend any group session	28(7.39)	12(3.20)	8(2.07)	48(4.21)
Attended one group session	3(0.79)	1(0.27)	1(0.26)	5(0.44)
Attended two group sessions	0(0.00)	2(0.53)	3(0.78)	5(0.44)
Attended three group sessions	3(0.79)	1(0.27)	1(0.26)	5(0.44)
Attended four group sessions	7(1.85)	0(0.00)	7(1.81)	14(1.23)
Attended five group sessions	5(1.32)	3(0.80)	6(1.55)	14(1.23)
Attended six group sessions	11(2.90)	11(2.93)	19(4.92)	41(3.60)
Attended seven group sessions	22(5.80)	19(5.07)	15(3.89)	56(4.91)
Attended eight group sessions	300(79.16)	326(86.93)	326(84.46)	952(83.51)
